# Screening and Treatment of Vitamin D Deficiency in UK Patients with Crohn’s Disease: Self-Reported Practice among Gastroenterologists

**DOI:** 10.3390/nu12041064

**Published:** 2020-04-11

**Authors:** Jane Fletcher, Amelia Swift, Martin Hewison, Sheldon C Cooper

**Affiliations:** 1 Nutrition Nurses, Queen Elizabeth Hospital Birmingham, University Hospitals Birmingham, Edgbaston, Birmingham B15 2TH, UK; 2 School of Nursing, Institute of Clinical Sciences, University of Birmingham, Edgbaston, Birmingham B15 2TT, UK; a.swift@bham.ac.uk; 3 Institute of Metabolism and Systems Research, University of Birmingham, Edgbaston, Birmingham B15 2TT, UK; m.hewison@bham.ac.uk; 4 Gastroenterology, Queen Elizabeth Hospital Birmingham, University Hospitals Birmingham, Edgbaston, Birmingham B15 2TH, UK; sheldon.cooper@uhb.nhs.uk

**Keywords:** Crohn’s disease, vitamin D deficiency, screening, clinical practice

## Abstract

Currently, there is no UK national recommendation to measure vitamin D levels in patients with inflammatory bowel diseases (IBD). Patients with IBD are at risk of developing vitamin D deficiency with the highest prevalence frequently reported in those with Crohn’s disease (CD). Treating vitamin D deficiency as part of CD management continues to be of interest. Our aim was to identify influences on practice and self-reported practice among British Society of Gastroenterology (BSG)-IBD section members in the screening and the treatment of vitamin D deficiency in patients with CD. A web-based survey was distributed via email to members of the BSG-IBD section. Reported screening practice was generally annual in those with a history of previous surgery related to CD or small bowel CD. A total of 83% of respondents (*n* = 64) thought that vitamin D levels should be routinely monitored in patients with CD. Treatments for mild/moderate deficiency included increased sunlight exposure (mean frequency = 21, SD = 15) and dietary advice (mean frequency = 22, SD = 14); in moderate/severe deficiency, oral supplementation was recommended (mean frequency = 14, SD = 13). Respondents reported factors most likely to influence practice, including clearer evidence and guidance. Well conducted studies in CD patients with identified vitamin D deficiency are needed to inform national guidance and clinical practice.

## 1. Introduction

It is recognised that patients with inflammatory bowel diseases (IBD) are at high risk of developing vitamin D deficiency [1], with the highest prevalence often reported in those with Crohn’s disease (CD) [2,3,4,5]. Though it remains unclear if this is a cause or a consequence of the disease, there is growing interest in the non-skeletal benefits of treating vitamin D deficiency in this group, as previously described by the authors [1]. Studies have suggested a correlation between vitamin D deficiency and poorer clinical status in IBD [2,6]. Higher or normalised levels of vitamin D in IBD have been correlated with improved response to anti-tumour necrosis factor-alpha medications [7] and a reduced risk of CD related surgery [8,9]. The National Institute for Health and Care Excellence (NICE) Clinical Guideline CG152 regarding management of CD [10] was updated and superseded in 2019 by NICE guideline NG129 [11] following review of the most current evidence available. Neither version of the guideline mentions vitamin D deficiency in this high-risk patient group. Currently, there is no UK national recommendation to monitor vitamin D levels in patients with CD, although standardised oral vitamin D and calcium supplementation is suggested where patients are receiving steroids for treatment of IBD [12]. In the same patient group, the European Crohn’s and Colitis Organisation (ECCO) [13] suggest supplementation to maintain vitamin D levels within the recommended range but do not stipulate what the range is. The American Gastroenterological Association (AGA) position statement suggests vitamin D and calcium supplementation, particularly in those at high risk of osteoporosis [14]. The British Society of Gastroenterology (BSG), ECCO, and AGA guidelines regarding osteoporosis in IBD do not advocate routine measurement of serum vitamin D levels [12,13,14]. Nevertheless, routine analysis of serum/plasma 25-hydroxyvitamin D (25OHD, the main circulating form of vitamin D) has become more widely available in recent years, with recent advances such as high throughput liquid chromatography-tandem mass spectrometry methods greatly improving reliability [15]. An alternative strategy of measuring calcium and parathyroid hormone (PTH) to detect secondary hyperparathyroidism, inferring vitamin D deficiency, has been suggested as a functionally more meaningful marker of vitamin D deficiency [12]. Whilst this approach provides a meaningful picture of functional vitamin D activity, it is not clear whether calciotropic responses to vitamin D can be extrapolated to anti-inflammatory function of vitamin D in IBD. Furthermore, the processing of blood samples for estimation of PTH levels is more laborious with rapid processing required. AGA suggest that PTH is only required in the event of abnormal calcium serum or urinary levels [14].

Despite the lack of clear national guidance, there is continued interest in the detection and the treatment of vitamin D deficiency as part of CD management. An understanding of influences and barriers to vitamin D screening in CD is warranted, including currently used methods for treatment of deficiency. This current practice survey explored clinicians’ self-reported clinical practice and knowledge. An overall impression of practice, rather than practice in specific age groups, was a key purpose, therefore no distinction was made between adult and paediatric practice. Members of the BSG-IBD section were eligible to take part in the survey within the defined study period. University of Birmingham ethical approval was gained (ERN_19-0128).

Our aim was to identify self-reported practice and influences in practice among BSG-IBD members in the UK in screening for and treatment of vitamin D deficiency in people with CD.

## 2. Materials and Methods

The study was a web-based survey designed using the Research Electronic Data Capture (REDCap) tools hosted at the University of Birmingham [16]. Study data were collected anonymously and managed using REDCap. The Strengthening the Reporting of Observational Studies in Epidemiology (STROBE) cross sectional reporting guidelines were used [17].

### 2.1. Survey Distribution

The survey link was distributed via group email by the BSG communications team to members of the IBD section in March 2019 and again approximately 2 weeks later. The survey was also advertised on the BSG “News” page of their website. The survey was open for 1 month. A covering email was sent as part of the group email distribution inviting members to participate (Supplementary Figure S1). The intention was to distribute to all members of the BSG-IBD to ensure that all registered members with a specific interest in CD had the opportunity to take part.

### 2.2. Survey Design

Survey questions were created to consider key areas of interest, including whether participants thought that vitamin D levels should be routinely measured in people with CD, what their usual practice was in terms of frequency of screening for vitamin D deficiency in CD, awareness of any guidelines related to vitamin D in people with CD, factors that might influence their decision to screen, factors that might influence their future practice, treatments they recommend or use in their practice for different levels of vitamin D deficiency if detected, and other nutritional monitoring and investigations into bone health they might routinely carry out. The authors hoped to gain an overall impression of clinician’s knowledge related to risk factors and management of vitamin D deficiency in this group. This might then be used to inform future national guidance.

The survey (S1) included a total of 20 questions plus 6 branching logic questions. This included multiple choice questions, free text questions, and some matrix questions as the most succinct way to present multiple options [18]. Overall time taken to complete the survey was anticipated to be 5–10 min. Features enabled within the data capture tool included the option to save and return, allowing greater flexibility in completing the survey.

### 2.3. Ethical Considerations—Consent

The survey was sent to healthcare professionals within their professional role, and therefore it was assumed that they had capacity to consent to participation. Participant information was provided at the start of the survey with the participant selecting agreement to participate when opening the survey. If the participant did not indicate their agreement to participate, the survey closed, and no data were collected.

### 2.4. Statistical Analysis

Data were exported from RED Cap to Microsoft Office Excel™ (Microsoft Corporation, Redmond, WA, USA) 2010 software for analysis of data and production of graphs. Results are presented using descriptive summary statistics, including percentages, frequencies, variance, and standard deviation (SD) for grouped data where appropriate. Qualitative responses were collated into themes for presentation.

## 3. Results

A total of 75 respondents agreed to participate in the survey. However, data were entered for only 64 respondents, with complete data in 62. Although the distribution list included up to 985 members, it was not possible to determine how many members actually received the email. The BSG communications team reported that the survey received approximately 200 clicks, indicating that this number of people had seen the email and opened it. Using click rate to determine number of people who received the email gave a response rate of 32%.

### 3.1. Demographic Data of Respondents

Table 1 shows the demographic data of respondents, including reported profession, institution, and age range. The majority of respondents were Gastroenterology Consultants.

Responses were received from all areas of the UK except Wales. The highest number of responses were received from participants in the Midlands (England) (19%), North West England (18%), and South East England (16%).

### 3.2. Vitamin D Screening in Practice

In total, 83% of respondents thought that vitamin D levels should be checked routinely in patients with CD. Of the 11 who thought that it should not be routinely measured, reasons given included: lack of guidance (*n* = 2) lack of evidence (*n* = 9), too expensive (*n* = 2), not necessary (*n* = 1), and not in trust guidelines (*n* = 1).

Table 2 shows the reported frequency of vitamin D screening by Crohn’s disease (CD) sub-type and CD treatment. Screening was most likely to be carried out annually and most often in those with small bowel CD and in those with a history of previous surgery related to their CD.

**Figure 1:**
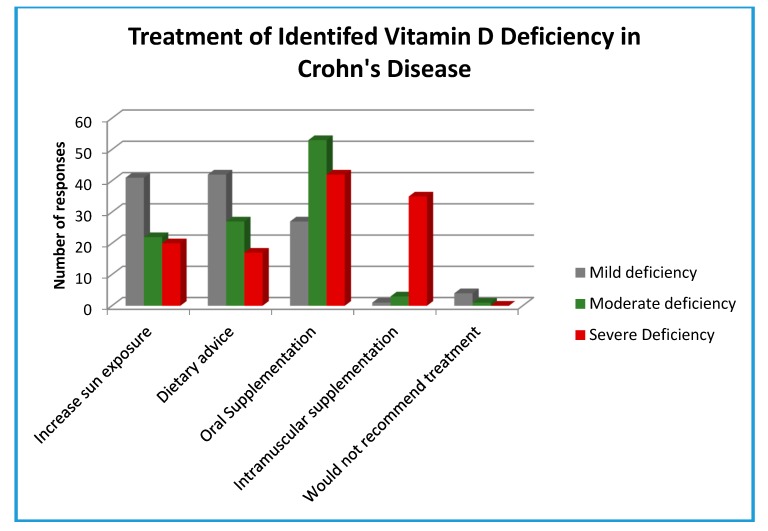
Treatment of identified vitamin D deficiency in Crohn’s disease. Vitamin D deficiency was classified as mild (35–49 nmol/L, moderate (15–34 nmol/L), and severe (<15 noml/L) in the survey.

Figure 1 shows the treatments respondents might recommend for identified vitamin D deficiency in CD. For the purposes of the survey, deficiency was classified as: mild (35–49 nmol/L, moderate (15–34 nmol/L), and severe (<15 noml/L). Increased sunlight exposure (mean frequency = 21, SD = 15) and dietary advice (mean frequency = 22, SD = 14) were most often recommended for those with mild to moderate deficiency. Oral supplementation (mean frequency = 14, SD = 13) was most often recommended for those with moderate to severe deficiency, whereas supplementation by intramuscular injection (mean frequency = 2, SD = 7) was predominately used by respondents in patients with severe deficiency.

### 3.3. Season, Ethnicity and Other Factors

Participants were asked if factors such as ethnicity or season were likely to influence their decision to carry out vitamin D screening in people with CD. A total of 23% (15/64) of respondents were more likely to check vitamin D levels in those with darker skin or Asian background. A total of 30% (19/64) of respondents reported that season may influence their decision to check vitamin D. Other factors that respondents considered were those with restricted diet/poor nutritional state (*n* = 2) and those with low sun exposure for any reason, including covering their skin (*n* = 6).

### 3.4. Awareness of Guidelines

Respondents reported awareness of a number of guidelines related to vitamin D and CD. These were ECCO (*n* = 11), BSG (*n* = 5), other osteoporosis guidelines, including those from the United States (USA) (*n* = 3), nutritional societies including UK, Europe, and USA (*n* = 1), local guidelines (*n* = 1), and NICE (*n* = 1).

### 3.5. Influences on Practice

Participants were asked what factors were most likely to cause them to change their practice in vitamin D screening. Responses included better clinical evidence (*n* = 25), clear guidance (*n*= 23), patient request (*n* = 23), if it was stipulated within trust guidelines (*n* = 8), and relevant education (*n* = 6). Two respondents reported that they would not change their practice.

### 3.6. Other Nutritional Monitorin

**Figure 2:**
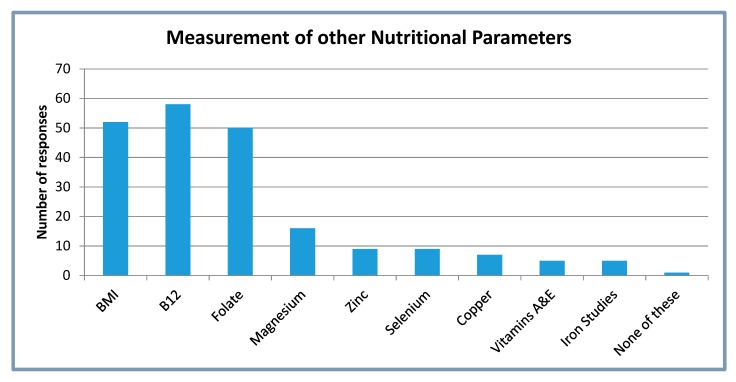
Measurement of other nutritional parameters in Crohn’s disease patients. BMI = body mass index. Iron studies includes ferritin.

Figure 2 shows other selected nutritional parameters commonly measured in patients with CD. Vitamin B_12_ and body mass index are most commonly measured.

### 3.7. Bone Health

Respondents reported that they would investigate bone health for the following reasons: recurrent steroid use (*n* = 57), malnutrition (*n* = 44), based on severity of CD (*n* = 34), based on duration of CD (*n* = 28), vitamin D deficiency (*n* = 28), and according to guidelines (*n* = 21). Other reasons included growth issues and based on sex, age, or menopause.

## 4. Discussion

The results of this survey suggest that the majority of respondents were carrying out vitamin D screening in patients with CD at least annually, despite the lack of clear national guidance. This was primarily in those with small bowel CD and those who had previous surgery related to CD. This could suggest that these sub-groups are considered to be at higher risk by respondents. It seems that respondents also considered additional risk factors for vitamin D deficiency, including ethnicity, season, lack of sun exposure, and poor nutritional status. Nutritional parameters were also commonly measured in patients with CD, including body mass index, vitamin B_12_, and folate. There was a recognition of the risks recurrent steroid use presents to bone health with 57 respondents reporting that they would investigate bone health further in this group.

The use of oral supplementation was most often implemented to treat moderate vitamin D deficiency, whereas increasing sun exposure and dietary advice were mostly recommended in response to mild deficiency. It is not clear from this survey how effective these treatments are in managing vitamin D deficiency in patients with CD. Currently, there is no clear national guidance regarding treatment or effective doses for vitamin D supplementation in CD. Current recommendations regarding vitamin D levels are based on skeletal function of vitamin D [1]. Within these recommendations, 25(OH) D levels of >50 nmol/L are considered adequate [1]. Recently, however, it has been suggested that, in terms of inflammatory markers and clinical indices, levels of 25(OH) D >75 nmol/L are optimal in patients with IBD [19,20]. Nielsen et al. [19] suggest a useful approach to vitamin D supplementation in patients with IBD, taking into account several factors, including disease activity, level of deficiency, and malabsorption. The results of this current survey suggest that some clinicians, at least, are already following a number of the strategies suggested by Nielsen et al. [19], including screening annually, considering increased sunlight exposure, and supplementation with oral or intramuscular preparations as required to manage vitamin D deficiency.

We believe that this is the only currently published survey that focuses specifically on clinician self-reported practice in vitamin D screening of people with CD in the UK. Wagnon et al. [21] reported an American survey of gastroenterologists’ awareness and implementation of the American Gastroenterological Association (AGA) guidelines on osteoporosis in IBD. Measuring vitamin D is recommended by AGA [14] in the detection of risk of osteoporosis, however, screening for vitamin D deficiency prior to supplementation is not covered within the survey [21].

The measurement of vitamin D serum levels is somewhat contentious, with one respondent to the current survey commenting that their institution did not allow them to measure serum vitamin D if the patient had a normal calcium level.

### 4.1. Survey Design

A number of factors were considered in the design of the survey to aid with participant response. A web-based survey delivered via email was chosen as a recognised and acceptable method for gathering information from healthcare professionals [22]. E-mail methodology has the advantage that it remains until actively deleted, thus it is less likely to be lost or mislaid than a paper based/postal survey. The survey was open for one month, giving participants adequate time to complete it. A single reminder was sent after approximately two weeks. This seemed to be a reasonable number of reminders within the given time, as excess reminders tend not to increase responses [23]. Furthermore, the survey was distributed via the BSG as a reputable, professional society relevant to the population.

A systematic review and meta-analysis (*n* = 20) found that survey length was significantly associated with response rate, with longer surveys having a poor response rate (*p* = 0.0001) [24]. However, the authors advise caution, as they note that the *p* value for test of homogeneity was *p* = 0.03, concluding that response rate cannot solely be attributed to survey length. Within our survey, survey length was considered, and the number of questions was kept purposely small to reduce the burden to participants.

### 4.2. Limitations

Despite these strategies, a key limitation of this survey was the distribution method. Using a third party to distribute the survey via group email ensured anonymity for participants. In addition, the third party, namely the BSG, is a respected professional society. The authors recognize that the number of responses received, in comparison to the number of members in the BSG-IBD group, may not be representative of the overall group. It is likely that those who responded were clinicians with an interest in vitamin D and, therefore, their practice may not reflect general gastroenterology practice across the UK. However, the authors had no control over the technical aspects of the distribution and therefore cannot determine with confidence how many members received the survey email. For this reason, the reported “click rate” (*n* = 200) was used to calculate the response rate of 32%.

This is within the average response rate of 30–35% anticipated for healthcare professionals [22] but significantly lower than other surveys distributed via the BSG to a similar audience. A UK web-based survey with eight questions regarding diathermy practice for colonic polypectomy had a 71.8% response rate [25]. A further European wide web-based survey contained 32 questions regarding the structure of training programmes on paediatric endoscopy [26]. This survey was also sent via a professional society (Young ESPGHAN) and had a response rate of 62% [26]. It appears that the main differences between the administration of these surveys and our current survey were the length of time the survey was open and the number of reminders sent. Despite this, it could be argued that the topic of the current practice survey was the prevailing factor impacting response rate.

Fan and Yan [27] suggest that the salience of survey content is one of the main influencing factors on response rate. The published surveys by Verma and Chilton 2019 [25] and Broekaert et al. 2019 [26] pertain to core skills in endoscopy required in the role of a gastroenterologist. An awareness only of osteoporosis is required in specific disease states, and vitamin D deficiency is not mentioned at all in the UK specialty curriculum [28]. It is possible that some clinicians did not find the topic of vitamin D deficiency in CD of interest or relevance to them. However, to validate this theory, a repeat of the current practice survey is required over a longer period of time and with multiple reminders. There have been other published surveys regarding practice in management of vitamin D deficiency amongst other healthcare professionals. An online survey of dietitians in Australia distributed to members of the Dietitian Association Australia via the weekly online state newsletter had only a 3% response rate [29].

## 5. Conclusions

It may be surmised that the respondents to our current practice survey were those clinicians with an active interest in vitamin D deficiency, with only 14% of respondents reporting that they rarely/never monitor vitamin D in CD. The key reasons cited for not monitoring vitamin D in this patient group were a lack of evidence and a lack of guidance. The key benefit of vitamin D is widely known to be with skeletal health. There are few randomised controlled trials (RCTs) investigating the general non-skeletal effects of vitamin D supplementation [30]. Only about a quarter of these include participants with an identified vitamin D deficiency [30]. In other disease states, vitamin D supplementation has shown to be of benefit, for example, in reducing the incidence of respiratory tract infections [31], breast cancer [32], and non-skin cancer in women aged over 55 years [33]. Furthermore, in women whose 25(OH)D concentrations were maintained ≥100 nmol/L, a 60% lower preterm birth risk was observed compared to those with 25(OH)D levels <50 nmol/L [34]. In terms of CD and IBD generally, evidence for vitamin D supplementation is mostly observational in nature [35], with some RCTs giving conflicting evidence [19].

The lack of definitive evidence leads to a dearth of clear clinical, national guidance and disparity in practice. Emerging evidence is revealing the consequences of vitamin D deficiency among those with CD, leading to increased frequency of anaemia, admission to hospital, surgical intervention, and incidence of colorectal cancer [1]. Yet, the questions surrounding the potential benefits to CD patients when vitamin D deficiency is both detected and treated remain unanswered. There continues to be a need for well conducted RCTs in patients with identified vitamin D deficiency, taking into account the principles set out by Heaney [36] in the design and the analysis of nutrient based clinical studies. In this way, clinical practice and national guidance may be informed.

## Figures and Tables

**Table 1:** Demographic Data of Respondents.

Demographic Data of Respondents	
Reported Profession	
Gastroenterology Consultants	*n* = 48 (77%)
Gastroenterology Registrars	*n* = 11 (18%)
Registered Nurses	*n* = 3 (5%)
Institution	
University Teaching Hospital	*n* = 36 (58%)
District General Hospital	*n* = 25 (40%)
Primary Care	*n* = 1 (2%)
Age Ranges (years) (Mean = 43 years, SD = 9 ± 75)	
30–39	*n* = 13 (21%)
40–49	*n* = 19 (31%)
50–59	*n* = 27 (43%)
60+	*n* = 3 (5%)

**Table 2:** Reported Frequency of Vitamin D Screening.

Sub-Type of Crohn’s Disease andTreatment	Reported Frequency of Vitamin D Screening in Crohn’s Disease *n* = 64			
	Annually *%*	6 monthly *%*	3 monthly *%*	Rarely/Never *%*
Small bowel CD	55	26	5	12
Crohn’s colitis	53	16	3	28
Peri-anal CD	41	16	3	41
				
Immuno-modulators	48	29	3	21
Biologic therapy	48	28	2	22
Steroids	44	27	13	16
History of surgery secondary to CD	56	23	7	15

## Supplementary Materials

The following are available online at https://www.mdpi.com/2072-6643/12/4/1064/s1, Figure S1 Vitamin D Screening in Crohn’s Disease: Current Practice Survey.
