# Supplementary material for: Screening and Treatment of Vitamin D Deficiency in UK Patients with Crohn’s Disease: Self-Reported Practice among Gastroenterologists
Source: Nutrients. 2020 Apr 11;12(4):1064. doi: 10.3390/nu12041064 (PMC7230977; doi:10.3390/nu12041064)
Supplement: Supplementary file 1 [file nutrients-12-01064-s001.zip › VitaminDScreeningInCrohnSDisea (1).pdf 16032019 V1.0.pdf]

Vitamin D Screening in Crohn's Disease: Current Practice Survey

Thank you for taking a few moments to read this information.

You are invited to take part in a short survey regarding your current clinical practice in vitamin D screening in patients with Crohn's Disease. This research is funded by the National Institute for Health Research (NIHR) through a Clinical Doctoral Research Fellowship.

The survey should take about 5 minutes however you can save your answers and return to complete it at a later time. To avoid duplication of data it is important that you only complete the survey once.

The survey closes on 15th April 2019.

Thank you

Participant Information and Consent

Introduction
The purpose of this research project is to identify your current practice in monitoring for vitamin D deficiency in patients with Crohn's Disease and commonly recommended treatments for vitamin D deficiency. The research is being conducted by Jane Fletcher at University Hospitals Birmingham NHS Foundation Trust as part of a National Institute for Health Research (NIHR) Clinical Doctoral Research Fellowship.

Why have I received this?
You are invited to participate in this research project because you are a member of the British Society of Gastroenterology - IBD group. While there is no direct benefit to you in taking part it is hoped that your expert opinion and experience in the field may be used to influence future research and treatments for patients with Crohn's Disease.

What does it involve?
The research involves completing an online survey that will take approximately 5-10 minutes. Your responses will be treated confidentially. The survey questions will be about your usual clinical practice in measuring vitamin D levels in patients with Crohn's Disease and treatment of vitamin D deficiency in this patient group. It is important that you complete the survey only once to avoid duplication of data.

Your participation in the survey is voluntary and you may choose to withdraw at anytime. The data you have provided prior to withdrawal will be used to inform the overall results of the survey. You will not be able to withdraw data once submitted. All data is stored in a password protected electronic format. The results of this study will be presented at professional conferences and meetings and published in relevant medical journals.

Confidentiality
We are not collecting any identifiable information during the survey.

Ethical approval
This research has received ethical approval from the University of Birmingham

Contact
If you have any queries regarding the survey please contact Jane Fletcher, Nutrition Nurse Lead, Queen Elizabeth Hospital Birmingham:
Telephone: 0121 371 4561 Email: jane.fletcher@uhb.nhs.uk.

Funding
This publication presents independent research funded by the National Institute for Health Research (NIHR) and Health Education England through a Clinical Doctoral Research Fellowship, Jane Fletcher ICA-CDRF-2017-03-083. The views expressed are those of the author(s) and not necessarily those of the NHS, the NIHR or the Department of Health and Social Care

○ I agree to participate in the survey

◯ I do not wish to participate in the survey

Vitamin D Screening

The following questions are about your usual practice in checking vitamin D levels for patients with Crohn's Disease.

1 Do you think vitamin D levels should be routinely checked in patients with Crohn's Disease? (select one)

◯ Yes
◯ No

1a Why do you think this? (select all that apply)

☐ Lack of guidance
☐ Lack of evidence
☐ Too expensive
☐ Not in my Trust guidelines/protocol
☐ Not necessary in this patient group
☐ Other

1b If 'Other', please specifiy

____

2 Approximately how often do you check vitamin D levels in patients with the following types of Crohn's Disease? (select one for each)

	3 monthly	6 monthly	Annually	Rarely or never
Small Bowel Crohn's Disease	◯	◯	◯	◯
Crohn's Colitis	◯	◯	◯	◯
Perianal Crohn's Disease	◯	◯	◯	◯

3 Approximately how often do you check vitamin D levels in patients with Crohn's Disease receiving the following treatment? (select one for each treatment)

	3 monthly	6 monthly	Annually	Rarely or never
Immuno-modulators	◯	◯	◯	◯
Biologic Therapy	◯	◯	◯	◯
Steroids	◯	◯	◯	◯
History of previous surgery due to Crohn's Disease	◯	◯	◯	◯

4 Are you aware of any guidelines related to monitoring vitamin D in patients with Crohn's Disease? (select all that apply)

☐ Local or Trust
☐ Regional
☐ National
☐ European
☐ Worldwide
☐ Not aware of any

4a Please specify any guidelines that you are aware of

____

5 Does the season determine whether you are more likely to check vitamin D levels in a patient with Crohn's Disease?

◯ Yes
◯ No

6 Does the patient's ethnicity determine whether you are more likely to check vitamin D levels in patients with Crohn's Disease?

◯ Yes
◯ No

6a Please give more detail for your answer: __________________________________

7 Are there any other cultural, religious or socio-economic factors that would influence your decision to check vitamin D levels in a patient with Crohn's Disease?

◯ Yes
◯ No

7a Please give more detail for your answer __________________________________

8 If you do not routinely monitor vitamin D levels in patients with Crohn's Disease what is most likely to influence your practice? (select all that apply)

☐ Clear guidance
☐ Better clinical evidence
☐ Stipulated in my Trust protocol/guidelines
☐ Relevant education
☐ Patient request
☐ National media
☐ I would not change my current practice
☐ Not applicable, I already monitor vitamin D levels

The following question is about your usual practice in recommending treatment for vitamin D deficiency.

At what level would you recommend the following as a treatment for vitamin D deficiency in patients with Crohn's Disease? (This might be different for different types of Crohn's Disease but consider Crohn's patients you see most commonly in your clinical practice)

	Mild deficiency (35-49 nmol/l)	Moderate deficiency (15 - 34 nmol/l)	Severe deficiency (< 15nmol/l)
Increase sunlight exposure	☐	☐	☐
Dietary advice to increase vitamin D derived from foods	☐	☐	☐
Oral vitamin D supplementation	☐	☐	☐
Intramuscular vitamin D supplementation	☐	☐	☐
I would not recommend treatment	☐	☐	☐

9a Please give more detail for your answer

____

The following questions are about any other types of nutritional monitoring you may carry out and investigations in to bone disease in patients with Crohn's Disease.

10 Do you routinely measure any of the following in patients with Crohn's Disease? (select all that apply)

☐ Body mass index (BMI)
☐ Fat soluble vitamins A and E
☐ Iron Studies including ferritin
☐ Folate
☐ Vitamin B12
☐ Zinc
☐ Copper
☐ Selenium
☐ Magnesium
☐ I don't check any of these

11 What would be a trigger for you to carry out investigations in to bone health, such as DEXA scan, in a patient with Crohn's Disease? (select all that apply)

☐ Vitamin D deficiency
☐ Duration of Crohn's Disease
☐ Severity of Crohn's Disease
☐ Presence of malnutrition
☐ Recurrent steroid use
☐ Guideline recommendation
☐ Other
☐ I would not investigate bone health

11a Please specify

____

12 Any other comments you would like to make?

The final section is about you.

13 What is your professional group? (select one from the dropdown box)

◯ Gastroenterology Consultant
◯ Gastroenterology Resigtrar
◯ Pathologist
◯ Radiologist
◯ Surgeon
◯ Registered Nurse
◯ Allied Health Professional

14 What is your age group?

◯ 20-29 years
◯ 30-39 years
◯ 40-49 years
◯ 50-59 years
◯ 60-69 years
◯ 70+ years

15 What geographical area is your usual place of work in the UK?

◯ Scotland ◯ North East England ◯ North West England ◯ Midlands (England) ◯ Wales ◯ London ◯ South East England ◯ South West England ◯ Northern Ireland ◯ I am based outside of the UK

16 In what setting is your usual place of work?

☐ University teaching hospital ☐ District General Hospital ☐ Primary Care ☐ University academic
